# Exploring the potential of the platelet membrane proteome as a source of peripheral biomarkers for Alzheimer's disease

**DOI:** 10.1186/alzrt186

**Published:** 2013-06-13

**Authors:** Laura E Donovan, Eric B Dammer, Duc M Duong, John J Hanfelt, Allan I Levey, Nicholas T Seyfried, James J Lah

**Affiliations:** 1 Department of Neurology and Center for Neurodegenerative Disease, Emory University School of Medicine, 615 Michael Street NE, Atlanta, Georgia 30322, USA; 2 Department of Human Genetics, Emory University School of Medicine, 615 Michael Street NE, Atlanta, Georgia 30322, USA; 3 Department of Biochemistry, Emory University School of Medicine, 1510 Clifton Road NE, Atlanta, Georgia 30322, USA; 4 Department of Biostatistics and Bioinformatics, Emory University School of Medicine, 1518 Clifton Road NE, Atlanta, Georgia 30322, USA

**Keywords:** blood biomarkers, platelet activation, mass spectrometry, membrane proteomics, glycoprotein, matrix metalloprotease inhibitor, amyloidogenic protein, alpha granule secretion, coagulation

## Abstract

**Introduction:**

Peripheral biomarkers to diagnose Alzheimer's disease (AD) have not been established. Given parallels between neuron and platelet biology, we hypothesized platelet membrane-associated protein changes may differentiate patients clinically defined with probable AD from noncognitive impaired controls.

**Methods:**

Purified platelets, confirmed by flow cytometry were obtained from individuals before fractionation by ultracentrifugation. Following a comparison of individual membrane fractions by SDS-PAGE for general proteome uniformity, equal protein weight from the membrane fractions for five representative samples from AD and five samples from controls were pooled. AD and control protein pools were further divided into molecular weight regions by one-dimensional SDS-PAGE, prior to digestion in gel. Tryptic peptides were analyzed by reverse-phase liquid chromatography coupled to tandem mass spectrometry (LC-MS/MS). Ionized peptide intensities were averaged for each identified protein in the two pools, thereby measuring relative protein abundance between the two membrane protein pools. Log_2_-transformed ratio (AD/control) of protein abundances fit a normal distribution, thereby permitting determination of significantly changed protein abundances in the AD pool.

**Results:**

We report a comparative analysis of the membrane-enriched platelet proteome between patients with mild to moderate AD and cognitively normal, healthy subjects. A total of 144 proteins were determined significantly altered in the platelet membrane proteome from patients with probable AD. In particular, secretory (alpha) granule proteins were dramatically reduced in AD. Of these, we confirmed significant reduction of thrombospondin-1 (THBS1) in the AD platelet membrane proteome by immunoblotting. There was a high protein-protein connectivity of proteins in other pathways implicated by proteomic changes to the proteins that define secretory granules.

**Conclusions:**

Depletion of secretory granule proteins is consistent with a preponderance of post-activated platelets in circulation in AD. Significantly changed pathways implicate additional AD-related defects in platelet glycoprotein synthesis, lipid homeostasis, amyloidogenic proteins, and regulators of protease activity, many of which may be useful plasma membrane-expressed markers for AD. This study highlights the utility of LC-MS/MS to quantify human platelet membrane proteins and suggests that platelets may serve as a source of blood-based biomarkers in neurodegenerative disease.

## Introduction

Alzheimer's disease (AD) is the most common form of dementia worldwide. Pathologically, it is characterized by the accumulation of extracellular beta amyloid (Aβ) plaques and intracellular tau tangles as well as gliosis and neuronal cell death [1-3]. More recently, abnormalities in synaptic transmission and vesicular trafficking have been reported in early AD [4,5]. As the population ages and the number of people affected with AD increases, it is becoming increasingly important to identify biomarkers that can be used to diagnose the disease as early as possible. While significant progress has been made in brain imaging and characterizing fluid biomarkers of AD in cerebrospinal fluid (CSF) [6,7], peripheral biomarkers have not been well established for clinical use. Blood-based biomarkers are especially attractive in a clinical setting compared to CSF, because blood samples are relatively easy to obtain.

Potential sources of blood-based biomarkers are platelets, small (1 to 4 μ), anuclear fragments derived from megakaryocytes in the bone marrow [8,9]. Platelets are dynamic and can exist in either a resting or activated state [8,9]. Resting platelets are inert; however, once activated, they undergo restructuring of their cytoskeleton and secrete numerous biologically active factors including cytokines, chemokines, and neurotransmitters [10]. Although activated platelets are perhaps best known for their role in hemostasis and thrombosis, they also play a significant role in inflammation and immunity [11]. Interestingly, platelets share many similarities with synaptic terminals in neurons and have been used as a model for studying synaptic vesicle metabolism. For example, both platelets and neurons secrete and respond to neurotransmitters and share many of the same secretory pathways and transporters for neurotransmitter uptake and packaging [12-14]. Platelets also contain a high concentration of amyloid precursor protein (APP) [15-17] and possess α, β, and γ-secretases [18], enzymes responsible for generating the Aβ peptide. Increased levels of activated platelets have been reported in patients with early AD compared to healthy, age-matched controls, and the platelet activation state has been positively correlated with the rate of cognitive decline measured by the mini mental status exam (MMSE) [19]. Subsequent studies have reported that patients with amnestic mild cognitive impairment (MCI) with elevated levels of activated platelets were at an increased risk of progression to AD within 3 years [20]. Although a majority of the published studies supports that activated platelets are higher in patients with AD compared to healthy controls [19-21], other studies [16,22] have also reported a decrease in platelet activity in AD. Thus, given the similarities between platelets and neurons and previously reported abnormalities in the platelet activation state in AD, platelets may serve as a valuable source of peripheral biomarkers in patients clinically defined with probable AD [23-25], while an inventory of proteins changing in platelets of AD patients may also provide mechanistic insight into their change in activation status.

Mass spectrometry (MS)-based proteomics has become an essential tool for the detection, identification, and quantification of protein biomarkers from complex mixtures including cells and tissue [26]. Proteomic techniques can provide certain advantages over transcriptomic approaches, for example in detecting protein loss due to secretion, although mRNA is maintained for translation in circulating platelets despite their anuclear status [27]. RNA changes in platelets have been reported in disease [28]. Whole platelet proteome and subproteomes have been profiled using liquid chromatography coupled with tandem mass spectrometry (LC-MS/MS) [10,29,30], however, an analysis of the platelet proteome from patients with AD compared to that of cognitively normal controls has been largely unexplored. Cytoskeletal proteins (for example titin, filamin and actin) represent the most abundant proteins in platelets, contributing to their rigid structure [10]. A drawback of data-dependent LC-MS/MS is an intrinsic bias toward sequencing the most abundant proteins in a sample that limits the detection of less abundant proteins that may be changing in disease [31]. Reducing the complexity of the sample before LC-MS/MS analysis is one way to circumvent this problem. Thus, enriching for the membrane subproteome prior to LC-MS/MS not only reduces the number of cytoskeletal proteins, but maximizes the likelihood of detecting less abundant cell-surface transmembrane proteins altered in disease. Another advantage of cell-surface platelet membrane biomarkers is their ability to serve as targets for probes in orthogonal diagnostic screening approaches including flow cytometry, which can be readily employed in a clinical setting.

Herein, we report a comparative analysis of the membrane-enriched platelet proteome between patients with mild to moderate AD and healthy, cognitively normal, control subjects. Following label-free quantification of 1,957 proteins in 1,009 homology groups using extracted ion intensity peptide measurements, 144 proteins were determined significantly altered in the platelet membrane proteome from patients with probable AD. Ontology annotation of altered proteins revealed specific pathways changing in AD and several that are specific to platelets. In particular, proteins encompassing the α-secretory granule pathway including α, β, and γ-chains of fibrinogen, thrombospondin-1 (THBS1), von Willebrand factor and fibronectin were dramatically reduced in AD. Of these, we confirmed THBS1 reduction in the AD platelet membrane proteome by immunoblotting. Platelets release α-granule contents when activated [8,32]. Thus, the major loss of α-secretory granule proteins observed in the AD membrane proteome is consistent with enhanced platelet activation, and maintenance of post-activated platelets in circulation. We discuss other pathways, including platelet glycoprotein synthesis, lipid homeostasis, membrane-associated amyloidogenic proteins, and regulators of protease activity, as each of these pathways were also significantly represented in the proteins found to be changing in AD platelet membrane proteome. Together, these data highlight the utility of LC-MS/MS to identify and quantify platelet membrane proteins isolated from humans and suggest that platelets may potentially serve as a useful source of blood-based biomarkers in neurodegenerative disease. Whether these biomarkers have diagnostic value in AD will need to be established in future longitudinal studies including a larger number of participants.

## Materials and methods

### Participant selection

Participants were selected from the Clinical Research in Neurology (CRIN) database at the Emory Alzheimer's Disease Research Center (ADRC). Institutional Review Board (IRB) approval was obtained from Emory University and all participants gave written informed consent before being included in this study. Seven controls and seven probable AD participants were enrolled in the study (Table 1), of which five of each group were pooled for proteomic analysis, matched as closely as possible for sex and age. Control subjects were selected based on a current MMSE score greater than 27 out of a total 30 points. Patients with mild to moderate AD all had a consensus diagnosis of probable AD and were selected based on having an MMSE score between 10 and 24. Participants were selected to be as similar as possible between groups with respect to age and were matched based on whether or not they were taking aspirin. Exclusion criteria included participants on clopidogrel (for example Plavix), those with conditions that could cause an increase in platelet activation level (a history of cancer within the past 5 years, autoimmune disorders, any acute illness, or chronic illnesses such as end-stage liver disease, end-stage renal disease requiring hemodialysis, and end-stage heart failure), and patients with bleeding disorders or other blood dyscrasias.

### Platelet isolation from whole blood

Whole blood (40 cc) was collected in acid citrate dextrose (ACD) using a 21 gauge butterfly needle. To help prevent platelet activation and aggregation, the tourniquet was removed after the initial needle stick and the first 5 cc of blood withdrawn was discarded. Platelet isolation from the remaining 35 cc of blood was adapted from Quereshi *et al. *[10]. Centrifugation times were optimized to maximize platelet yield and purity based on analysis of the sample by light microscopy after each step. Blood was centrifuged at 200 × *g *for 20 minutes immediately after collection to separate red and white blood cells from platelet-rich plasma. The top 2/3 of the platelet-rich plasma was collected to minimize white blood cell contamination, and transferred to a 5 ml polypropylene tube. The platelet-rich plasma was kept at room temperature and centrifuged within three hours at 120 *g *x 6 minutes (Eppendorf 5810 centrifuge, Eppendorf AG, Hamburg, Germany) to remove additional remaining red and white blood cells. A majority (top 2/3) of the purified platelet-rich plasma was transferred to a second 5 ml polypropylene tube and centrifuged at 1500 × *g *for 10 minutes. The platelet-poor plasma was removed and the platelet pellet was resuspended in 1 ml of citrate wash buffer (11 mM glucose, 128 mM NaCl, 4.3 mM NaH_2_PO_4_, 4.8 mM sodium citrate, 2.4 mM citric acid, pH 6.5) and recentrifuged at 120 × *g *for 4 minutes. The washed platelets were transferred to an Eppendorf tube and pelleted at 1500 × *g *for 10 minutes in an Eppendorf 5417C table-top centrifuge. The platelet pellet was frozen at -80°C in citrate wash buffer to minimize *in vitro *platelet activation. Platelet purity was assessed using flow cytometry (Becton Dickinson LSRII digital benchtop analyzer, Becton, Dickinson and Co., Franklin Lakes, NJ, USA). Briefly, the sample of purified platelets was stained with allophycocyanin (APC)-tagged CD45 to identify white blood cells and fluorescein isothiocyanate (FITC)-tagged anti-CD41/integrin αIIβ to identify platelets. Data were analyzed using FlowJo (version 7.6.1) software (Tree Star Inc., Ashland, OR, USA).

### Membrane enrichment strategy

The membrane enrichment strategy employed was modified from previously published methods [33,34]. Briefly, frozen platelets were thawed on ice, resuspended in a hypotonic solution containing 100 μl citrate wash buffer and 900 μl deionized water, and kept on ice for 1 hour. Following hypotonic lysis, the mixture was sonicated (Sonic Dismembrator, Thermo Fisher Scientific, Waltham, MA, USA) twice for five seconds at 20% amplitude (maximum intensity) to disrupt cell membranes and large cytoskeletal fragments. Following sonication, the whole platelet homogenate (W) was centrifuged at 1500 × *g *for 10 minutes (Eppendorf 5417C) to sediment any cellular debris. The supernatant (S1) was transferred to a polycarbonate ultracentrifuge tube and centrifuged at 180,000 × *g *for one hour at 4°C (Beckman Optima TLX ultracentrifuge, TLA 100.4 rotor, Beckman Coulter Inc., Brea, CA, USA). The supernatant (S2) containing the soluble protein fraction was removed and saved. The resulting pellet (P2) was resuspended in 1 ml of 0.1 M sodium carbonate, pH 11 with protease and phosphatase inhibitors and incubated on ice for 15 minutes to strip proteins only loosely associated with the membrane. The samples were recentrifuged at 180,000 × *g *for one hour at 4°C (Beckman Optima TLX ultracentrifuge, TLA 100.4 rotor). The supernatant (W1) was removed and saved and the resulting membrane-enriched, insoluble pellet (P3) was dissolved in 50 μl 8 M urea 10 mM Tris pH 7.8. Protein concentrations from each of the five fractions (W, S1, S2, W1, P3) were determined by the bicinchoninic acid (BCA) method (Pierce, Rockford, IL, USA). The different fractions obtained from the enrichment protocol were analyzed by silver stain. Briefly, protein (1 μg) was loaded from each fraction into a 10% acrylamide gel and separated by gel electrophoresis. The gel was fixed in a solution containing 50% methanol and 5% acetic acid for 10 minutes and washed with deionized water. After rinsing in 0.02% sodium thiosulfate for 1 minute, the gel was stained with 0.1% silver nitrate for 10 minutes and developed with 3% sodium carbonate, 0.05% formaldehyde solution until the bands were sufficiently stained.

### Mass spectrometry, peptide identification and quantification

Protein (20 μg/case) from the membrane-enriched fraction (P3) was pooled for proteomic analysis. After pooling, samples were alkylated with 10 mM dithiothreitol (DTT) and 50 mM iodoacetamide (IAA). Total protein was loaded into a 10% acrylamide gel and separated by SDS-PAGE. Gels were stained with Coomassie blue overnight. After destaining, gel lanes were cut into three molecular weight regions. Individual gel regions were diced into 1 mm^3 ^pieces and destained with 50% acetonitrile (ACN) and 50 mM ammonium bicarbonate until the pieces became clear. Gel slices were digested overnight with trypsin (12.5 ng/μL; Promega Corp., Madison, WI, USA) diluted 1:20 in 50 mM NH_4_HCO_3 _at 37°C. The following day, peptides were extracted with buffer (5% formic acid, 50% ACN), dried in a SpeedVac concentrator (Thermo Scientific) and stored at -20°C. Purified peptides were analyzed by reverse-phase liquid chromatography coupled with tandem mass spectrometry (LC-MS/MS) and each sample was analyzed in technical replicate [35]. Briefly, peptide mixtures were loaded onto a C_18 _column [100 μm internal diameter (i.d.), 20 cm long, 2.7 μm HALO resin from Michrom Bioresources, Inc., Auburn, CA, USA] and eluted over a 10 to 30% gradient (Buffer A: 0.1% formic acid, 0.005% heptafluorobutyric acid, and 5% acetonitrile; Buffer B: 0.1% formic acid, 0.005% heptafluorobutyric acid, and 95% acetonitrile) for 90 minutes. Eluates were monitored in a MS survey scan followed by 10 data-dependent MS/MS scans on an LTQ-Orbitrap ion trap mass spectrometer (Thermo Finnigan, San Jose, CA, USA). The LTQ was used to acquire MS/MS spectra (3 m/z isolation width, 35% collision energy, 5,000 AGC target, 200 ms maximum ion time). The Orbitrap was used to collect MS scans (300 to 1600 m/z, 1,000,000 AGC target, 1,000 ms maximum ion time, resolution 30,000). All data were converted from raw files to the .dta format using ExtractMS version 2.0 (Thermo Electron, San Jose, CA, USA) and searched against human reference database downloaded from the National Center for Biotechnology Information (19 November 2008) using the SEQUEST Sorcerer algorithm (version 3.11, SAGE-N Research, San Jose, CA, USA). Searching parameters included mass tolerance of precursor ions (±50 ppm) and product ion (±0.5 m/z), partial tryptic restriction, with a dynamic mass shift for oxidized Met (+15.9949), two maximal modification sites and a maximum of two missed cleavages. Only b and y ions were considered during the database match. To evaluate the false discovery rate (FDR), all original protein sequences were reversed to generate a decoy database that was concatenated to the original database (77,764 entries) [36]. The FDR was estimated by the number of decoy matches (nd) and total number of assigned matches (nt). FDR = 2*nd/nt, assuming mismatches in the original database were the same as in the decoy database [36]. To remove false positive matches, assigned peptides were grouped by a combination of trypticity (fully and partial) and precursor ion-charge state (+2, +3 and +4). Each group was first filtered by mass accuracy (20 ppm) and by dynamically increasing correlation coefficient and ΔCn values to reduce theoretical protein FDR by the above measure to less than 1%. All MS/MS spectra for proteins identified by a single peptide were manually inspected as described previously [37]. If peptides were shared by multiple members of a protein family, the matched members were clustered into a single group. On the basis of the principle of parsimony, the group was represented by the protein with the greatest number of assigned peptides. All identified proteins (represented by the top homolog within a group (1,009 proteins identified with unique peptides) and ungrouped (1,957 total potentially identified proteins and isoforms)) are provided respectively in Table S1 and Table S2 in Additional File 1. Quantification of peptides and proteins was based on the comparison of paired peptides from AD and control samples. Ion current intensities for identified peptides were extracted in MS survey scans of high-resolution and a ratio of the peak intensities for the peptide precursor ion was calculated using in-house DQuan software as described previously [38].

### Establishing candidate biomarkers with statistical and pathway analysis

Statistical analysis to evaluate the significance of the protein changes was performed as previously described with modifications [38,39]. Relative differences in protein levels were derived from extracted ion intensities for all identified peptides and expressed as signal-to-noise ratios. A ratio of ion intensities for the peptide precursor ions from AD and control pools were calculated, log_2 _transformed, and averaged to obtain a protein ratio across samples (AD/control). A null experiment was represented by a comparison of log_2_-transformed protein ratios for control replicates (replicate 1/replicate 2). As predicted by the central limit theorem, the histogram of the differences (AD/control) and the null experiment (replicate 1/replicate 2) between protein log_2 _ratios fit Gaussian distributions, which enabled evaluation of systematic bias according to the mean and biological variation based on standard deviation (SD). A Gaussian curve for the binned frequencies of log_2 _ratios was determined using Igor Pro v6.1 (WaveMetrics, Inc., Lake Oswego, OR, USA) and the mean was subtracted from all log_2 _ratios to center the population of average AD/control log2 ratio and control 1/control 2 at zero. The SD was determined via the Gaussian width as 0.72 log_2 _ratio units for AD/control and 0.30 for the null experiment (control 1/control 2). A total of 144 proteins were considered significantly changed and met the following criteria, i) fell outside the null experiment distribution or beyond 99.9% confidence interval (3.29-fold SD), ii) had an absolute value ≥1.17, (3.55 times the standard deviation of the null experiment), iii) had a coefficient of variation (CV) of less than 100% and iv) had a signal-to-noise ratio greater than 10 in both control measurements. True biomarker FDR was estimated by counting the false positives surviving the above filters in the null experiment and calculating this number as a percentage of the total number of biomarkers proposed in the list for the AD/control comparison, as described in results. The list of gene symbols for these proteins was input into DAVID pathway analysis v6.7 [40], and significantly changing ontological classes of proteins were further considered in the context of available references which related each class to AD.

### Antibodies

Primary antibodies used in these studies were as follows: FITC-conjugated CD41/integrin αIIb (1:1000, mouse monoclonal (SZ.22); Abcam, Cambridge, MA, USA); APC-conjugated CD45 (1:1000, mouse monoclonal; BD Pharmingen, Franklin Lakes, NJ, USA); THBS1 (1:1000, mouse monoclonal; Thermo Fischer Scientific, Waltham, MA, USA); beta-actin (1:1000, mouse monoclonal; Abcam, Cambridge, MA, USA); the antibody dilutions for THBS1 and beta-actin reflect prior dilution of each antibody (1:1) with glycerol.

### Immunoblotting

Equal concentrations of protein from each sample were loaded into a 10% acrylamide gel and separated by SDS-PAGE. Proteins were transferred onto polyvinylidene fluoride (PVDF) Immobilon-P membranes (Millipore, Billerica, MA, USA) overnight at 4°C. Immunoblots were blocked for 2 hours at room temperature with Tris-buffered saline (TBS)/Tween and blocking buffer (5x blocking buffer ultrapure from US Biological, Salem, MA, USA) and probed for the protein of interest with a primary antibody overnight at 4°C. The following day, blots were incubated with fluorophore-conjugated secondary antibodies (1:20,000) for 1 hour in the dark. All blots were scanned and quantified using the Odyssey Infrared Imaging System (Li-Cor Biosciences, Lincoln, NE, USA). Statistical analysis was performed using a two-tailed Student's *t*-test.

## Results and discussion

### Participant selection

Characteristics of participants with clinically diagnosed AD and controls are presented in Table 1. Controls were selected to be as similar as possible to AD patients (matched as closely as possible with regard to age and sex). As expected, there was a significant difference between the groups in MMSE scores (*P *= 0.01). Aspirin status was matched between groups to help control for any effect of aspirin on the platelet proteome. By matching for aspirin usage, we were able to obtain a sample more representative of the general population affected by AD. Furthermore, ideal biomarkers will change in disease independent of factors such as medications. Apolipoprotein (Apo)E genotype of selected cases was not considered, since subsequent pooling of samples has the effect of cancelling specific differences in protein abundance due to individual case variation. Quantitative proteomic analysis found no significant difference in ApoE levels between control and clinically diagnosed AD platelet membrane fractions, as described below.

### Platelet isolation and membrane protein enrichment strategy

**Figure 1:**
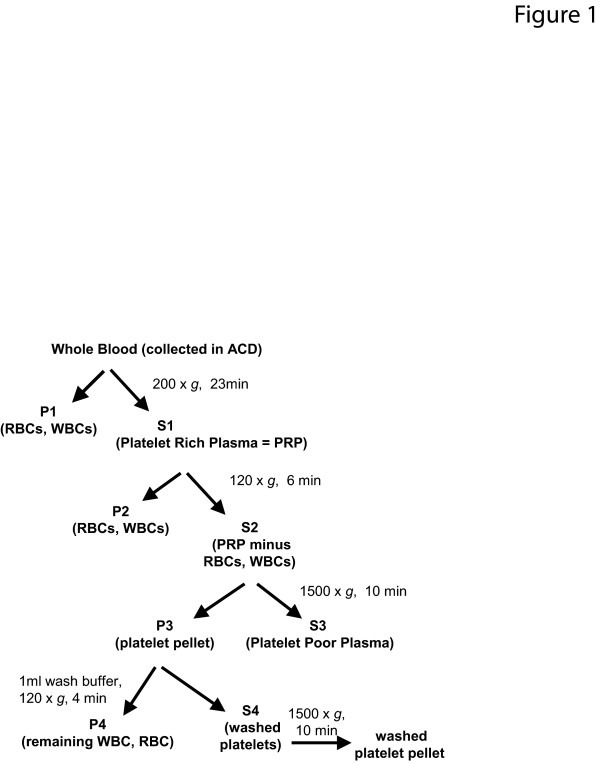
**Workflow diagram of platelet membrane purification protocol**.

**Figure 2:**
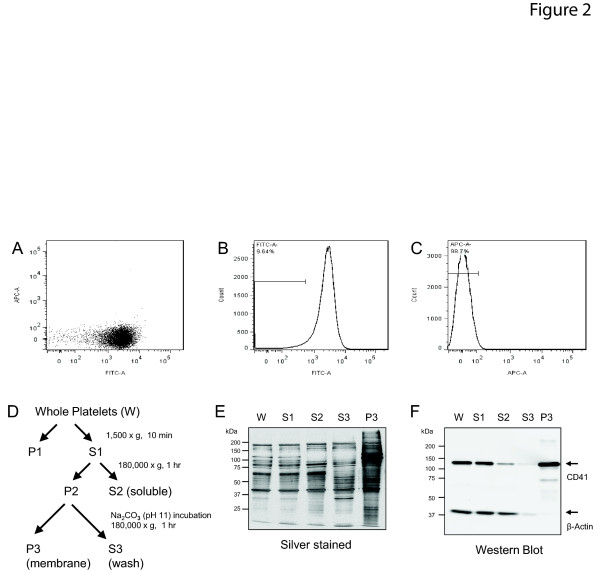
**Platelet isolation strategy yields samples >90% pure platelets by flow cytometry**. **(A) **Purified platelets were double stained for allophycocyanin (APC)-conjugated, anti-CD45 (white blood cell marker, y-axis) and fluorescein isothiocyanate (FITC)-conjugated anti-CD41 (platelet marker, x-axis). Double scatterplot demonstrates the majority of the sample stains positive for CD41, but not for CD45. **(B) **Histograms for CD41^+ ^(91.36%) flow cytometry events consistent with platelet-specific enrichment, and **(C)**, CD45^+ ^(1.3%) events. **(D) **Differential centrifugation workflow for enrichment of the membrane proteome from platelets isolated from whole blood, prior to LC-MS/MS analysis. **(E) **Silver stain of fractions described in panel D. (**F**) Immunoblot demonstrating CD41 enrichment and actin depletion during the workflow to produce platelet membrane fraction (P3). LC-MS/MS, liquid chromatography coupled to tandem mass spectrometry.

Platelets from whole blood were isolated through centrifugation as previously published [10] using a citrate buffer, which significantly minimizes *in vitro *platelet activation (Figure 1). To assess the purity of the isolated platelets, flow cytometry was performed after double-labeling with antibodies against the platelet-specific marker, CD41 (integrin αIIβ), and a marker for white blood cells, CD45. Results demonstrate that the platelet-enriched samples contained greater than 90% CD41 positive cells, whereas CD45 positive cells made up 1.3% of the cells (Figure 2A-C). Platelets contain an extensive intracellular membrane, an open canalicular system that serves as a reservoir for plasma membrane proteins and membrane receptors and provides a passage for granule release after activation [41]. There are also numerous membrane-bound granules in platelets, the contents of which could be more easily identified in a membrane-enriched sample. Differential centrifugation fractions obtained during enrichment of the membrane proteome prior to LC-MS/MS analysis (Figure 2D) were first visualized by silver stain of a representative sample indicating altered protein complexity in the whole (W), soluble (S2), wash (W) and membrane fraction (P3) (Figure 2E). Immunoblotting with antibodies against CD41, a platelet-specific transmembrane protein, demonstrated an average of 2.2-fold enrichment in the membrane fraction (Figure 2F). Conversely, immunoblotting with antibodies against actin, a cytoskeleton protein, demonstrated approximately 20-fold depletion in the membrane fraction compared to whole platelet lysate (averaged from three independent experiments). To assess the global enrichment of proteins in the membrane fraction, LC-MS/MS analysis was performed from equal amounts of whole platelet lysate (W) and membrane-rich fraction (P3). TMHMM 2.0 [38,42] was used to predict the number of transmembrane domains (TMD) for each protein. In the membrane-enriched fraction, 40% (389/966) of proteins were predicted to have a TMD. This was a 2.5-fold increase from an analysis of the whole platelet proteome performed in parallel in which 17% (225/1290) of proteins identified contained a predicted TMD (Table S2 in Additional file 1). The results are consistent with a similar previously published enrichment strategy for membrane proteins from platelets [30]. The most significantly enriched and depleted proteins in the membrane fraction were determined by relative quantification using spectral counting (Table S2 in Additional file 1). These included CD41 and beta-actin, which were significantly enriched and depleted, respectively, in the membrane fraction consistent with immunoblot analysis. Together, these results indicate that the differential centrifugation approach was effective at both enriching membrane proteins and depleting soluble cytoplasmic proteins.

### Label-free quantification of membrane-enriched proteome differences in AD

**Figure 3:**
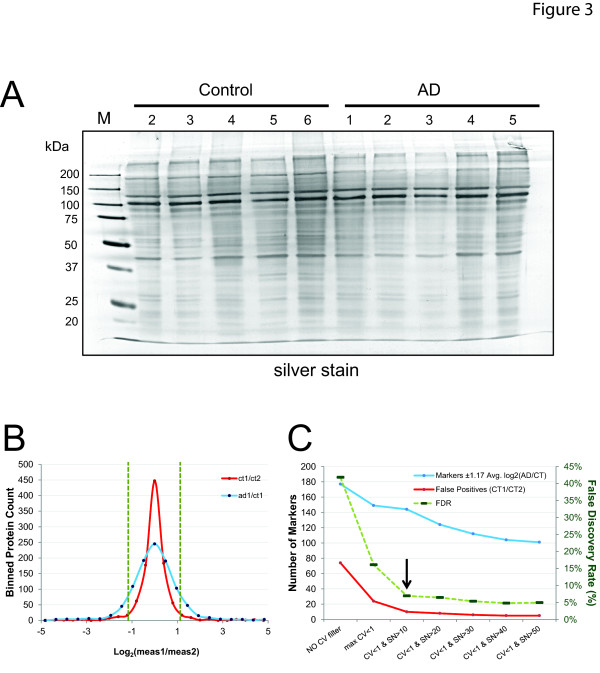
**Case-specific platelet membrane protein pooling and population results from quantitative proteomics**. **(A) **Five control (C) and five probable Alzheimer's disease (AD) case membrane proteomes that made up the control and AD platelet membrane pools were visualized by silver staining to confirm equal contributions to the pool and to demonstrate comparable purity and integrity. **(B) **Gaussian fits of the null experiment (red curve) and experimental comparison replicate one (blue curve) for the population of 1,009 quantified proteins binned according to log_2_-transformed ratio quantified relative abundance. Significance cutoff at ±1.17 is shown as dashed vertical lines. **(C) **False positive counts (red trace), candidate marker proteins (blue trace), and effect on false discovery rate (FDR) (dashed green trace, *scale to right*) of various filtering criteria. FDR was 6.9% at the selected criteria of 1.17 log_2_-transformed AD/CT ratio (average of two technical replicates), coefficient of variance <100%, and signal-to-noise minimum control level greater than 10.

To determine differences between AD and control membrane samples, pooled control or AD cases were analyzed by LC-MS/MS (Table 1). Pooling samples prior to LC-MS/MS analysis has been shown to decrease intersubject variability and enhance the likelihood that any changes detected would be universal to disease [43]. Prior to pooling, each control and probable AD membrane-rich protein fraction was visualized by silver staining following 1D gel electrophoresis to confirm equal protein contributions and to demonstrate comparable purity and integrity (Figure 3A). Peptides were extracted from the samples following an in-gel tryptic digest and analyzed in technical replicate using LC-MS/MS in a data-dependent manner as described in the methods. After database searching, we identified and quantified 7,910 peptides representing 1,957 proteins (Table S2 in Additional file 1, organized by homology groups with highest to lowest total number of peptide identifications). A total of 1,009 homologous protein groups (each with unique peptides that could be used for quantification) were identified across control and AD samples (Table S1 in Additional file 1, sorted by log_2 _(AD/control) fold difference from decreasing in AD to increasing). Of these, 38% (378/1009) contain at least one transmembrane domain predicted by TMHMM 2.0 [38,42], and DAVID ontology analysis reported that 55% (559/1009) had Protein Information Resource annotations relating to 'membrane'.

To determine candidate AD platelet membrane protein biomarkers from our list of 1,009 quantified proteins, we employed an approach to estimate true FDR that fully utilizes the power of technical replicates and a null experimental comparison to quantify false positives under any given filtering criteria [39]. Relative differences in protein levels, ion intensities for identified peptides, expressed as signal-to-noise ratios, were extracted in MS survey scans of high-resolution. A ratio of ion intensities for the peptide precursor ions from AD and control LC-MS runs were calculated, log_2 _transformed, and averaged to obtain a protein ratio across samples (AD/control), and a null experiment log_2_-transformed ratio for control replicates (replicate 1/replicate 2). As predicted by the null hypothesis, the histogram of the differences (AD/control) and null experiment between protein log_2 _ratios fit Gaussian distributions, which enabled us to evaluate systematic bias according to the mean and biological variation based on SD (Figure 3B). The null experiment has a much smaller SD (log_2 _= 0.30) than the average log_2 _(AD/control) population (SD = 0.72). This is consistent with high reproducibility across replicates and indicates that our quantitative bioinformatics approach has sufficient precision to detect the biological variance, which manifests as a much wider SD for the latter population. As a filtering criterion, proteins with potentially increased or decreased abundance in AD that fell outside the 99.9% two-tailed confidence interval were considered as a subgroup of interest. Increased confidence in the average of two technical replicates was obtained by restricting proteins considered significantly changed to those with a coefficient of variation (standard deviation as a percentage of the mean) of less than 100%, where this filtering criterion alone reduced false positives surviving filtering in the null experiment from 74 to 24 (Figure 3C). Further applying an additional filter for minimum signal-to-noise resulted in false positives dropping to 10 when a minimum signal-to-noise ratio of 10 was required. This translates to a FDR of 6.9%. The list of 144 significantly changed proteins corresponding to this FDR in AD relative to the control platelet membrane fraction is given in Table S3 in Additional file 1.

### Changes in platelet secretion and activation observed in patients with AD

Ontologies (categorization of the list into pathways, molecular functions, keywords, cellular compartments, and so on) significantly overrepresented within the list of 144 significantly changing proteins were determined using DAVID [40] (Table 2). Fifteen proteins, or about 10% of the list, represent factors likely specific to platelets that fall into the following six overlapping categories (A to F): platelet activation (Group A; *P *= 0.0029), platelet alpha granules (Group B; *P *= 5.1 × 10^-9^), secretory granules (Group C; *P *= 1.7 × 10^-5^), the complement control module (Group D; *P *= 0.012), complement and coagulation cascades (Group E; *P *= 2.3 × 10^-5^), and platelet alpha granule lumen (Group F; *P *= 1.8 × 10^-7^). All but one of the proteins in these six categories were significantly decreased, rather than increased, in AD relative to the control pool, including α-, β-, and γ-chains of fibrinogen. Fibrinogen is involved in the coagulation cascade and is secreted by alpha granules after platelet activation. It has also been included in several panels of biomarkers for AD. According to Thambisetty *et al*., decreased fibrinogen in association with other changes in plasma has been associated with lower brain volumes in AD [44]. Craig-Shapiro *et al*. have included fibrinogen in a multiplex immunoassay panel to analyze CSF biomarkers for AD. They reported that a finding of increased fibrinogen levels in the CSF in association with changes in other proteins increases the ability of the CSF tau/Aβ_42 _ratio to discriminate between patients with very mild to mild dementia and those who are cognitively normal [45]. Platelets release alpha granules when activated. As this study looked at a membrane-enriched fraction, this finding suggests that AD platelets have a generally decreased or exhausted reserve of alpha granules consistent with having undergone activation. We speculate that low levels of fibrinogen observed in platelets from patients with AD is complementary to the reported increase in fibrinogen infiltration into AD central nervous system (CNS) tissue associated with Aβ depositions and microglial activation [46]. Contact of platelets with amyloid aggregates has been shown to result in their activation [47], and Aβ stimulates abnormal clots of cleaved fibrinogen (fibrin) resistant to clearance [48]. These findings in combination suggest widespread AD-specific platelet activation, supported by previous studies that have reported platelet activation in individuals with AD [19-21].

The single increasing protein in Table 2, platelet glycoprotein IX (GP9), a surface protein on platelet and alpha granule membranes [49] is known to act as a receptor for von Willebrand factor [50]. This represents a novel platelet surface-expressed candidate marker that could be specifically increasing in a manner linked to AD. Surprisingly, other members of the GP9-containing transmembrane receptor complex, which has a reported stoichiometric configuration involving glycoproteins V and Ib alpha and beta chains [51], were well quantified and found to be unchanging in the AD platelet membrane proteome (Table S3 in Additional file 1). This suggests a change in the configuration of the multimeric receptor and potentially, a change in the responsiveness of platelets in AD individuals to von Willebrand factor. It is interesting to note that von Willebrand factor is well expressed in brain vascular endothelia [52]. Should an increase in GP9 correspond with an increase in platelet affinity for CNS vascular endothelial walls, this could be consistent with a causative role for increased surface GP9 on platelets in producing conditions whereby local von Willebrand factor and amyloid in CNS blood vessel endothelium stimulate alpha granule release and local fibrinogen invasion into the CNS of AD patients [46]. This hypothesis relies on the above findings and assumption, which await further validation in a broader cohort. In the remaining sections of this report, we discuss the broader subset of potential platelet membrane biomarkers found changing in probable AD beyond evidence for platelet activation, and possible insight they provide into disease mechanisms.

### Validation of a decrease in platelet thrombospondin-1 (THBS1) and AD-associated changes detected in amyloidogenic proteins

THBS1 is a large, homomultimeric extracellular matrix glycoprotein with multiple signaling functions in different cellular contexts. It is secreted from platelets, and also from astrocytes in the CNS, where it may stimulate neuronal synaptogenesis [53]. In the context of platelet membranes, THBS1 promotes thrombosis in at least two ways: (1) it stimulates platelet aggregation through CD36 receptor-based inhibition of kinase signaling cascades [54], and (2) THBS1 acutely counteracts the promotion of blood flow by nitric oxide via binding to another receptor, CD47, on vascular smooth muscle cells [55,56]. The platelet receptor CD36 was well quantified in the membrane proteome pools and found to be trending down (log_2 _(AD/control) -0.48, Table S3 in Additional file 1), though not significantly.

**Figure 4:**
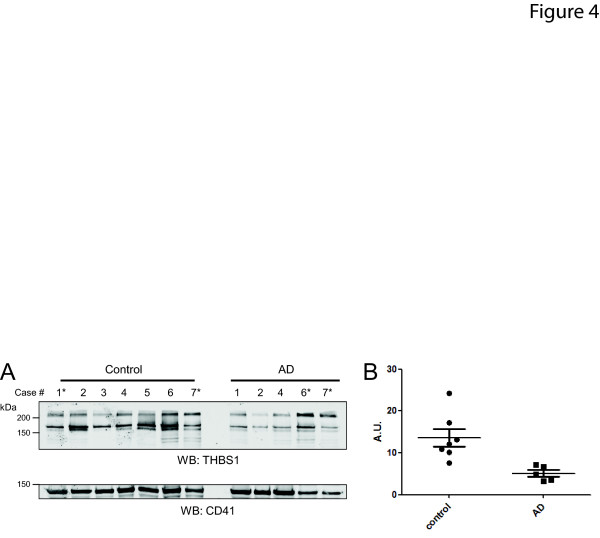
**Validation of thrombospondin-1 (THBS1) loss in Alzheimer's disease (AD) platelet membranes by immunoblot**. **(A) **Immunoblot of THBS1 and CD41 as a loading control for seven control, and five AD individual cases. Cases which contributed to the pools used for proteomics have no asterisk (*). **(B) **Densitometry analysis of the immunoblot in panel A.

To validate the potential AD-associated decrease in THBS1, the platelet membrane fraction from individual cases was immunoblotted with an antibody against THBS1. Validation of individual cases following proteomic analysis of pooled samples is important because sample pooling opens up the possibility that a large change in one individual could be driving the signal measured [38], despite the fact that interindividual variability generally is muted by pooling. In the pooled proteome quantitative analysis, THBS1 was decreased 75% in AD (log_2 _(AD/control) -2.02) and immunoblotting confirmed this result (*P *= 0.0085, Figure 4). Notably, some of the cases used for validation were not included in the proteomics analysis. However, the confirmation of decreasing THBS1 across a number of individuals with clinically diagnosed AD increases the likelihood that the decrease in THBS1 observed by proteomics for the AD pool is disease specific.

Reduced THBS1 in AD platelet membranes could be consistent with complementary evidence for secreted THBS1 in CNS. Buée *et al*. found that THBS1 stained senile plaques in AD brains and suggested it may be involved in plaque formation [57]. Recently, Horn *et al*. examined the effect of human neutrophil alpha-defensins, components of the innate immune system, on platelet activation. They found that these defensins activated platelets and led to fibrinogen and THBS1 binding. Moreover, these fibrinogen and THBS1 complexes formed amyloid-like structures. Such a cascade could also play a role in AD pathogenesis [58].

Other significantly changed amyloidogenesis-associated proteins identified in the platelet membrane proteome included increased beta-2 microglobulin (B2M, log_2 _(AD/control) 1.21) and decreased gelsolin (log_2 _(AD/control) -1.40). Increased B2M binding to the surface of blood cells including granulocytes, lymphocytes and monocytes is characteristic of chronic hemodialysis, and co-occurs with vascular and renal amyloid deposits of this protein [59]. Notably, none of the patients involved in this analysis had end-stage renal disease or required dialysis. Consistent with a specific effect of AD on this protein, elevated B2M was reported as one of eight CSF biomarkers, which together made up a multianalyte profile that was able to distinguish both probable AD and Parkinson's disease individuals from controls [60]. Earlier, high B2M in probable AD patient CSF was also found via a proteomic approach [61].

Gelsolin is a chaperone with multiple functions that has been shown to bind to Aβ [62] and ApoE [63] and has an independent involvement in certain amyloidoses. Although it is reportedly unchanging in AD brain, it was previously identified as a plasma AD marker that correlated positively with rapidity of cognitive decline in clinically diagnosed AD patients [64]. However, by itself, a decrease in plasma gelsolin is also associated with multiple morbidities including oxygen imbalances, major trauma, malaria, and liver injury [65]. Thus, although the changes we describe for amyloidogenic proteins including THBS1, B2M, and gelsolin in the platelet membrane proteome in AD are consistent with what is known to occur in individuals diagnosed with AD, it is also apparent that alone, these protein changes are not markers with adequate specificity for AD - obviating their inclusion into broader multianalyte profiles that consider a panel of changing proteins, be it on the membranes of platelets, or in CSF.

### Co-occurrence of other pooled analyte changes consistent with previous biomarker studies

Beyond the above potential markers for clinically diagnosed AD, which confirm platelet activation plus a change in each of three amyloidosis-linked proteins THBS1 (down), B2M (up), and gelsolin (down), we asked what other changes found are consistent with previously proposed AD markers or potentially linked to proteins involved in disease mechanism, albeit not necessarily through activity in platelets. By expanding this list, the results from the current study might be applied toward the development of a future blood test that utilizes a broad multianalyte profile to aid in the confirmation or diagnosis of AD with higher specificity and accuracy. In the list of 144 significantly changing proteins in AD platelet membrane fractions, we found five additional proteins that have been identified as potential biomarkers or have a function homologous to such a protein (Table 3). Mannosyl-glycoprotein acetylglucosaminyltransferase (MGAT) 4B, elevated 5.5-fold in the AD platelet membrane pool, is involved in extended glycosylation of proteins. Comparatively low expression of a functional homolog, MGAT3, was recently reported to distinguish a fraction of AD patients from controls [66]. A vacuolar protein sorting (VPS) 13C allele specified by a single intronic SNP was recently found to significantly co-occur with AD [67], and we found that there was a significant, 67%, decrease in the AD platelet membrane pool. Synthesis of an abundant membrane lipid class called plasmalogen has been found to be defective in AD, and the rate-limiting enzyme alkylglycerone phosphate synthase (AGPS) was found to be reduced in postmortem-confirmed AD brain [68]; in the platelet membrane pool in this study, AGPS was also significantly decreased, by 68%. Ferritin heavy and light chains, usually found in a 1:1 stoichiometry, increase with age in normal, but not AD brain, and a distinguishing feature of frontal cortex in AD compared to Parkinson's disease was a large, 5-fold, increase in heavy/light ferritin ratio [69]. Ferritin light chain AD/control ratio was significantly decreased nearly 4-fold (74%) in the pooled probable AD platelet membrane proteome. Finally, insulin signaling has been linked to AD pathogenesis in multiple studies, where insulin-like growth factor 1 receptor (IGF1R) expression and signaling decreases in AD brain. IGF1R signaling has been shown to reverse amyloid beta toxicity, perhaps via regulation of amyloid precursor cleavage [70]. IGF1R also significantly decreased 74% in the AD platelet membrane pools. In conclusion, the platelet membrane proteome harbors a rich pool of analytes, a number of which are changed significantly in clinically diagnosed AD and moreover in the case of some potential AD platelet-derived markers, these proteins changed consistent with previous measurements.

### Ten classes of potentially novel AD biomarkers quantified in platelet membrane pools, and the case for two additional platelet biomarker candidates

Following analysis of the 144 consistently changing proteins using DAVID bioinformatics, we manually curated 10 ontological classes of potentially novel AD markers in platelets (Table S4 in Additional file 1), where these class terms (numbered below) were found in searches of existing literature to be extensively linked to AD or CNS function, and to each other. For example, a hypothesis for calcium (1) dysregulation in AD has been reviewed [71], and related to mitochondria (2) dysfunction in AD [72]. Endocytic trafficking (8), including clathrin-mediated (7) and other forms of endocytosis (6), has been linked to amyloid beta toxicity in a recently published comprehensive yeast screen [73]. Myosin motor proteins (5) are important for neuronal vesicle transport (8) [74,75]. N-linked glycosylation (9) mediated by the isoprenoid lipid dolichol is dysregulated in AD [76,77], thereby implicating changes in glycoproteins (10) more generally as relevant. A loss in proteasome (3) function has been linked to various neurodegenerative conditions. While an AD-specific frontal cortex ubiquitin linkage profile did not implicate a general loss of proteasome function in AD [78], it is implicated in AD via an essential role for proteasomal degradation in modulating both inflammatory signaling outside of platelets and the degradation of tau in neurons following ubiquitination, which may be antagonized by tau phosphorylation promoted by Aβ [79,80]. Significant decreases in two pairs of interacting proteasome subunits copurifying with the membrane fraction were reliably quantified. Finally, platelets possess the capacity to undergo apoptotic cell death, and a loss of antiapoptotic factors (4), like that seen in the membrane proteome pool from platelets, could potentially precede neuronal loss during the course of AD.

Although we cannot review all the evidence linking the above classes or individual proteins to AD as potential proteins of mechanistic relevance or as biomarker candidates, one protein of interest in the platelet membrane fraction is reversion-inducing cysteine-rich protein with kazal motifs (RECK), which is decreased 91% in AD patients compared to controls. RECK is an inhibitor of matrix metalloprotease (MMP) proenzyme activation, including MMP2 [81] and MMP9 [82], but most interestingly, of the presumed alpha secretase APP cleavage enzyme ADAM10 [83]. The MMP2 and 9 extracellular matrix proteases have a prominent role in angiogenesis, but were once hypothesized to function as either alpha or beta secretases [84] and MMP9 has been proposed as a biomarker for CNS inflammation in early AD [85]. In CNS, MMP2 and MMP9 may have differential activity or localization, providing different opportunities for the degradation of Aβ. MMP9 is produced by CNS neurons and degrades Aβ [86], perhaps combating amyloid plaque accumulation, albeit at the cost of increased neuroinflammation [87]. Previously reported differences in plasma MMP2 versus MMP9 activity in AD [88] might have functional implications in whole blood only in the context of decreased platelet RECK and THBS1, which has also been reported to act as an effective inhibitor of the same MMPs [89].

A second and final example of a distinguishing protein likely bound to the surface of platelet membranes is ApoB, an important component of very low-density lipoprotein (VLDL) particles and chylomicrons, which transport postprandial triglycerides from intestine to the liver. Although no significant change occurred in other platelet-associated lipoproteins, including ApoA1 (log_2 _(AD/control) -0.09), ApoE (log_2 _(AD/control) 0.54), ApoO-like (log_2 _(AD/control) -0.68) or ApoJ (clusterin, log_2 _(AD/control) -0.64), ApoB was decreased 72% (log_2 _(AD/control) -1.86) in the AD platelet membrane fraction. ApoB is a highly polymorphic protein with two forms. The mRNA of the B100 form is posttranscriptionally edited at a single base to change a glutamine-encoding codon to nonsense, resulting in a shorter B48 form [90]. An artificial mutation that only produces the B100 form lowers cholesterol levels [91] while the B48 form enriches VLDL particles with high triglyceride levels [92]. The LDL receptor binding site is determined downstream of the B48 stop codon, as determined by an R3500Q mutation in B100 that decreases LDL particle affinity for its receptor [93]. The initial report of mRNA editing also demonstrated that the expression and activity of the specific RNA editase is promoted by insulin [90]; hyperinsulinemia is a major risk factor for AD [94] and has also been linked to an increase in cognitive markers of premature brain aging in individuals without AD [95]. Upon close examination, the decrease in platelet-associated ApoB measured was driven by peptides encoded exclusively by the B100 mRNA, which are encoded after the editase-dependent stop codon at residue 2180 (data not shown). This does not rule out a general decrease in ApoB binding to platelets, where THBS1 (previously described as a significantly decreasing protein) is one of a number of platelet proteins capable of binding to both VLDL and chylomicrons [96]. However, existing evidence for elevated ApoB-48 co-occurring with high Aβ in the intestinal enterocytes that serve as the normal site for ApoB RNA editing and secretion of B-48 containing chylomicrons [97] lends support for the potential usefulness of the ApoB-48/ApoB-100 ratio associated with platelets as a potential biomarker, which should be further explored, in parallel with the alternate possibility that pan-ApoB association with platelets could be decreased. Furthermore, evidence implicates that ApoB-containing lipoprotein particles can strongly influence the activity of prothrombotic proteases [98,99].

**Figure 5:**
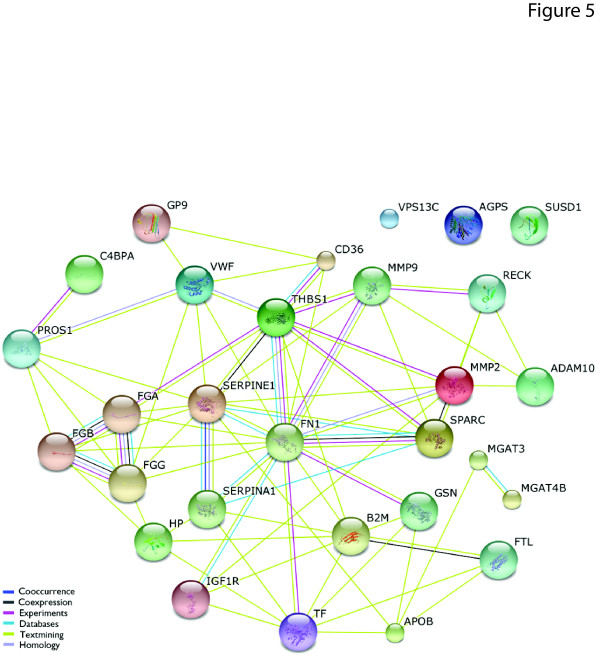
**Functional interactions among proteins discussed in this study as potential biomarkers for Alzheimer's disease (AD) in platelet membrane proteome**. STRING [108] v9.0 was used to map functional interactions among the 15 platelet-activation associated proteins listed in Table 3 and other proteins mentioned as potential biomarkers.

Throughout the discussion of our results, it is notable that the platelet membrane proteome changes are often functionally linked to the process of thrombosis. To visualize the best-established functional interactions of the putative biomarkers discussed throughout these results, we built an interaction network (Figure 5). Strikingly, most of the potential biomarkers uncovered indeed do have established functional linkage to the tightly integrated multi-hubbed network of alpha granule components.

## Conclusions

In this study, we purified platelet membrane proteins for quantitative proteomics and identify potential biomarkers and pathways affected in patients with clinically diagnosed AD. In line with previous findings, many of the platelet-specific pathways that are changing are involved in platelet activation, and this is consistent with a role for Aβ peptide in activating platelets and leading to platelet aggregation [47]; moreover, APP from platelets is a major source of Aβ in circulating blood [15,100], suggesting a potential feed-forward mechanism since APP is established to be an alpha granule component [101], and its mobilization via platelet activation could lead to increased circulating Aβ. We did not sequence any APP Aβ peptide in the extracted membrane proteome, although APP was sequenced by eight peptides distributed across residues 41 to 662 in the total platelet proteome, suggesting that amyloid processing may occur *in vitro *during or prior to the membrane enrichment process and consistent with the presence in platelets of the full complement of secretase activities [18]. Alternatively or in addition, platelet activation, or decreased platelet activity in AD [22,102], may coincide with variable control of vascular risk factors in patients across studies. Vascular risk factors that can coincide with platelet activation include diabetes, hypertension, hypercholesterolemia, and/or atherosclerosis [103,104]. In this small study, matching controls to AD individuals for medication use was performed only for aspirin. Thus, it is possible that other vascular risk factors not sufficiently controlled by medications, could thereby affect platelet activation. Vascular risk factors are established to increase the risk of developing AD or promoting AD progression [105,106] which reasons that variability in the acute or chronic presentation of these factors may coincide with variable disease progression. Ideally, future studies should measure the stability of the platelet membrane proteome between consecutive blood donations to quantify intra-subject variation, whereas the measurement of inter-subject variability would require proteomic comparisons across individual, rather than pooled cases.

Although our findings indicate a broad set of potential AD biomarkers occurring among proteins associated with platelet membranes, it is important to cite inherent constraints. Glycoproteins and proteins with high hydrophobicity or with multiple transmembrane domains can be underestimated following trypsin digestion [107]. However, both AD and control pools were prepared similarly and peptide intensities were directly paired and compared by our bioinformatics approach. Therefore, this minor limitation mainly hampers abundance comparisons across different proteins, and estimation of absolute protein amount, which were not necessary for our determination of candidate differential biomarker status. However, the first major limitation of our study is small sample size. A much larger and more diverse sample would be required before drawing any definitive conclusions about platelet differences co-occurring with AD. Second, all of the cases in this study were clinically diagnosed, and as such are probable AD cases; diagnostic errors occur in about 5 to 10% of cases based on postmortem pathological confirmation from brain tissue. While it is possible that one or more patients in this study could have a form of dementia (for example vascular dementia) other than AD, a diagnosis of probable AD was given only when no other cause of dementia was likely based on patient presentation, past medical history, CSF biomarker studies for tau and Aβ, and neuroimaging results. All of these patients received a consensus diagnosis of AD from a group of board certified neurologists who specialize in dementia. Third and finally, additional validation of the specificity of platelet markers for AD would require inclusion of additional out-groups from patients with other types of dementia as well as patients with conditions that cause platelets to be activated (such as sepsis or cancer, or any of the isolated vascular risk factors described above in isolation from cognitive impairment status).

Despite the above caveats, this study provides unique insight into pathways changing in platelets in individuals diagnosed with AD. We have presented findings that evoke insights into existing literature and provide evidence for platelet membrane-associated proteins as potentially useful disease markers which co-occur in the periphery or possibly even derive from active mechanisms of disease progression or prognosis. These markers could be part of a predictive multianalyte profile with the potential to be determined via future blood-based tests that are both specific and accurate with regard toward confirming diagnosis of probable AD.

## Abbreviations

Aβ: beta amyloid; ACD: acid citrate dextrose; ACN: acetonitrile; AD: Alzheimer's disease; AGPS: alkylglycerone phosphate synthase; APC: allophycocyanin; Apo: apolipoprotein; APP: amyloid precursor protein; B2M: beta-2 microglobulin; BCA: bicinchoninic acid; CSF: cerebrospinal fluid; CNS: central nervous system; CV: coefficient of variance; DTT: dithiothreitol; FDR: false discovery rate; FITC: fluorescein isothiocyanate; GP9: glycoprotein IX; IAA: iodoacetamide; IGF1R: insulin-like growth factor 1 receptor; LC-MS/MS: liquid chromatography coupled to tandem mass spectrometry; MCI: mild cognitive impairment; MGAT: mannosyl-glycoprotein acetylglucosaminyltransferase; MMP: matrix metalloprotein; MMSE: mini mental status exam; MS: mass spectrometry; PAGE: polyacrylamide gel electrophoresis; PVDF: polyvinylidene fluoride; RECK: reversion-inducing cysteine-rich protein with kazal motifs; SD: standard deviation; SNP: single nucleotide polymorphism; TBS: Tris-buffered saline; THBS1: thrombospondin-1; TMD: transmembrane domains; (V)LDL: (very) low density lipoprotein; VPS: vacuolar protein sorting.

## Competing interests

The authors declare that they have no competing interests.

## Authors' contributions

LED, DMD and NTS performed the experiments. EBD, DMD and NTS analyzed and compiled data. LED and EBD prepared the manuscript with input upon revision from all authors. Reagents and resources were provided by AIL, JJL and NTS, who also collaboratively designed the experiments. All authors read and approved the final manuscript.
