# Correction to: Exploring the potential of the platelet membrane proteome as a source of peripheral biomarkers for Alzheimer’s disease

**DOI:** 10.1186/s13195-021-00839-y

**Published:** 2021-05-18

**Authors:** Laura E. Donovan, Eric B. Dammer, Duc M. Duong, John J. Hanfelt, Allan I. Levey, Nicholas T. Seyfried, James J. Lah

**Affiliations:** 1 Department of Neurology and Center for Neurodegenerative Disease, Emory University School of Medicine, 615 Michael Street NE, Atlanta, GA 30322 USA; 2 Department of Human Genetics, Emory University School ofMedicine, 615 Michael Street NE, Atlanta, GA 30322 USA; 3 Departmentof Biochemistry, Emory University School of Medicine, 1510 Clifton Road NE, Atlanta, GA 30322 USA; 4 Department of Biostatistics and Bioinformatics, Emory University School of Medicine, 1518 Clifton Road NE, Atlanta, GA 30322 USA

**Correction to: Alz Res Therapy 5, 32 (2013)**

**https://doi.org/10.1186/alzrt186**

It has recently come to our attention that a paper we published in 2013 in Alzheimer’s Research & Therapy never included the tables (Tables 1, 2 and 3) and supplemental tables (Additional file 1) in the published manuscript [1]. This must have been an oversight at the proof or production stage.

Tables 1, 2 and 3 are shown below.

**Table 1:** Participants

Controls			Probable Alzheimer’s Disease (AD)		
Case #	Age	MMSE^a^	Case #	Age	MMSE^a^
1	75	28	**1**	**83**	**20**
**2**	**72**	**29**	**2**	**77**	**18**
**3**	**74**	**30**	**3**	**90**	**11**
**4**	**67**	**29**	**4**	**83**	**20**
**5**	**73**	**29**	**5**	**82**	**24**
**6**	**60**	**30**	6	61	12
7	69	30	7	55	25
AVERAGES:	70.00	29.3		75.86	18.6

Cases in bold were pooled for proteomic analysis

Bold type-face indicates cases pooled for proteomic analysis

^a^*MMSE* mini mental status exam, clinical measurement of cognitive function

**Table 2:** DAVID analysis of changes in proteins associated with platelet-specific secretion

Mean log_**2**_ (AD/CT)	R1 vs R2 CV, SD%	Symbol	Protein Name	Ontology Groups
**−1.70**	**0.6%**	FGA	fibrinogen alpha chain	A, B, C, E, F
**−2.03**	**7.5%**	FGB	fibrinogen beta chain	A, B, C, E, F
**−1.69**	**1.5%**	FGG	fibrinogen gamma chain	A, B, C, E, F
**−2.02**	**1.9%**	VWF	von Willebrand factor	A, B, C, E
**−1.23**	**39%**	PROS1	protein S (alpha)	B, C, E, F
**−1.19**	**59%**	FN1	fibronectin 1	B, C, D, F
**−1.19**	**41%**	SERPINA1	serpin peptidase inhibitor, clade A (alpha-1 antiproteinase, antitrypsin), member 1	B, C, E
**−1.47**	**31%**	SPARC	secreted protein, acidic, cysteine-rich (osteonectin)	B, C, F
**−2.05**	**0.9%**	THBS1	thrombospondin 1	B, C, F
**1.47**	**1.2%**	GP9	glycoprotein IX (platelet)	B, C
**−2.82**	**2.9%**	C4BPA	complement component 4 binding protein, alpha	D, E
**−1.24**	**94%**	SERPINE1	serpin peptidase inhibitor, clade E (nexin, plasminogen activator inhibitor type 1), member 1	E, F
**−1.83**	**66%**	TF	transferrin	C
**−1.65**	**10%**	HP	haptoglobin-related protein; haptoglobin	D
**−1.33**	**31%**	SUSD1	sushi domain containing 1	D

**Table 3:** Overlap of AD platelet membrane protein changes with previously proposed mechanistic and diagnostic biomarkers

Mean log_**2**_ (AD/CT)	R1 vs R2 CV, SD%	Symbol	Protein Name
2.46	14%	MGAT4B	mannosyl (alpha-1,3-)-glycoprotein beta-1,4-N-acetylglucosaminyltransferase, isozyme B
−1.23	52%	VPS13C	vacuolar protein sorting 13 homolog C (*S. cerevisiae*)
−1.24	20%	AGPS	alkylglycerone phosphate synthase
−1.92	15%	FTL	ferritin, light polypeptide
−1.97	7.0%	IGF1R	insulin-like growth factor 1 receptor

## Supplementary Information

**Additional file 1: Supplementary Table S1.** 1009 proteins identified by unique peptides (top group members) identified in platelet membrane proteome from either a probable AD pool or a cognitive normal pool. **Supplementary Table S2.** 1957 total proteins (all potential group members) identified in platelet membrane proteome from either a probable AD pool or a cognitive normal pool. **Supplementary Table S3.** 144 potential marker proteins identified in platelet membrane proteome from a probable AD pool (FDR < 7%). **Supplementary Table 4.** 10 Classes incorporating 97 potentially novel AD biomarkers quantified in platelet membrane pools.
